# Association between geospatial disparities in food security with weight loss and nutritional outcomes of metabolic surgery

**DOI:** 10.1007/s00464-024-11175-1

**Published:** 2024-08-21

**Authors:** Avia D. Wilkerson, Corey Gentle, Elizabeth N. Dewey, Nitin Sajankila, R. Blake Buchalter, Andrew T. Strong, Xiaoxi Feng, Mary Elizabeth Patti, Sofya Asfaw, Rickesha Wilson, Ali Aminian

**Affiliations:** 1 Department of General Surgery, Cleveland Clinic Foundation, 9500 Euclid Avenue, A100, Cleveland, OH 44195 USA; 2 Center for Populations Health Research, Department of Quantitative Health Sciences, Cleveland Clinic, Cleveland, OH USA; 3 Research Division, Joslin Diabetes Center, Harvard Medical School, Boston, MA USA; 4 Cleveland Clinic Lerner College of Medicine, Cleveland, OH USA

**Keywords:** Food security, Food insecurity, Metabolic surgery, Bariatric surgery, Sleeve gastrectomy, Gastric bypass, Nutrition

## Abstract

**Background:**

Food insecurity has been linked to higher rates of obesity. It has also been shown to diminish the effectiveness of weight loss strategies, including intensive lifestyle interventions. One essential component of food insecurity is having a geospatial disadvantage in access to healthy, affordable food, such as living within a food desert. This study aims to determine if food insecurity also impacts weight loss and nutritional outcomes in patients who underwent Roux-en-Y gastric bypass (RYGB) or sleeve gastrectomy (SG).

**Methods:**

Clinical outcomes of patients who underwent RYGB or SG at Cleveland Clinic or affiliate regional hospitals in the United States from 2010 to 2018 were collected. Modified Retail Food Environmental Index (mRFEI) data was collected from the Center for Disease Control and merged with patient census tract data, allowing the patient cohort to be divided into those living in areas identified as food secure (mRFEI > 10%), food swamps (mRFEI = 1–10%), or food deserts (mRFEI = 0). Postoperative weight change was evaluated with quadratic growth mixture models and stratified by surgery type.

**Results:**

A total of 5097 patients were included in this study cohort, including 3424 patients who underwent RYGB and 1673 who underwent SG. The median duration of follow-up was 2.3 years (IQR 0.89–3.6 years). Food security status was not associated with postoperative weight change (RYGB *p* = 0.73, SG *p* = 0.60), weight loss nadir (RYGB *p* = 0.60, SG *p* = 0.79), or weight regain (RYGB *p* = 0.93, SG *p* = 0.85). Deficiencies in nutritional markers at 1–2 years after surgery were also not significantly different between food security groups.

**Conclusion:**

Despite the established relationship between food insecurity and obesity, food insecurity does not negatively impact weight loss or nutritional outcomes following RYGB or SG, demonstrating metabolic surgery as a powerful and equitable tool for treating obesity.

**Level of Evidence:**

IV.

Obesity is a serious chronic condition of increasing prevalence [1]. Prior studies have linked higher rates of obesity with residing in food deserts due to barriers prohibiting access to healthy food. A food desert is defined by the U.S. Department of Agriculture (USDA) as an “area characterized by relatively poor access to healthy and affordable food” [2]. Others have linked high densities of supermarkets within 0.5 miles of one’s residence to lower body mass index (BMI) [3]. Local groups have identified multiple food deserts with the Cleveland metropolitan area as well as within broader Cuyahoga County. These locations coincide with historically redlined areas, with markedly increased rates of poverty. Prior collaboration between state, county and city initiatives with boards of health have indicated that greater than 59% of all Cleveland residents and 35% of all Cuyahoga County residents live in food deserts, which have disproportionate representations of non-white residents [4].

Metabolic surgery has proven to be the most efficacious and durable intervention for weight loss and metabolic syndrome remission in patients with severe obesity [5]. Several studies have demonstrated that decreased adherence to the recommended post-metabolic surgery diet can be associated with weight regain and nutritional deficiencies. While poor self-discipline and lack of motivation can be intrinsic factors associated with poor diet adherence after metabolic surgery, limited availability of healthy foods has been suggested as an important extrinsic factor [6].

Previous studies have shown negative associations between food insecurity and successful weight loss interventions in patients with obesity. Myers et al. found that food insecurity modulates weight loss following high intensity lifestyle-based weight loss interventions, with decreased weight loss in patients facing lack of sufficient healthy food sources [7]. A limited number of studies have examined the impact of poor access to nutritious foods on weight loss and nutritional outcomes following metabolic surgery [8]. This study aims to compare postoperative outcomes of patients who underwent metabolic surgery by utilizing geospatial data to determine if patients who reside in food insecure areas experience reduced weight loss and/or worse nutritional outcomes than patients who reside in food secure neighborhoods.

## Materials and methods

### Data description

This is an Institutional Review Board-approved retrospective cohort study in which data were queried from our institutional contributions to the Metabolic and Bariatric Surgery Accreditation and Quality Improvement Program (MBSAQIP) to identify patients 18 years or older who underwent sleeve gastrectomy (SG) or Roux-en-Y gastric bypass (RYGB) within Cleveland Clinic hospitals in Ohio from 2010 to 2018. Patients who underwent a revision or conversion surgery, and patients who did not live in Ohio or whose census tract could not be identified were excluded (*n* = 1243). Body weight on all postoperative visits across four follow-up years was exported from the electronic health record and merged with preoperative MBSAQIP data. Census tracts for individual patients were identified using patient addresses at the time of surgery. Modified Retail Food Environmental Index (mRFEI) data was collected from the Center for Disease Control (CDC) and merged with patient data by census tract. The mRFEI is a measure which indicates the percentage of healthy food retailers within a census tract or ½ mile from the tract boundary relative to all food retailers within the area [9]. It has been widely used across studies which have established relationships between food insecurity and various outcomes such as glycemic control [10], obesity rates [11], and physical activity [12]. Healthy food retailers include supermarkets, larger grocery stores, supercenters, and produce stores. Less healthy food retailers include fast food restaurants, small grocery stores, and convenience stores [9].

### Study groups

Patients were divided into one of three food security groups based on the mRFEI of the census tracts in which they resided at the time of surgery. The CDC defines mRFEI scores of 0% as food deserts (F_Desert_). A food swamp (F_Swamp_) is defined by mRFEI scores from 1–10% in other publications. Food swamps correspond to areas where convenience stores or fast food restaurants represent most of the available food with little fresh produce available. The final group, with mRFEI scores greater than 10% is designated as Food Secure (F_Secure_). These are areas where more than 10% of food outlets offer healthy food options and fresh produce.

### Weight loss outcomes

BMI was calculated based on weight and height data extracted from the electronic medical record. Preoperative BMI was assessed from patient weight recorded on the day of the index metabolic surgery. Given maximum weight loss is usually achieved in the first few years after metabolic surgery [13], BMI nadir was calculated as the lowest postoperative BMI achieved within four years post-surgery. BMI regain was calculated as the maximum postoperative BMI following BMI nadir within the four-year time frame following the index metabolic surgery. The time to achieving BMI nadir was calculated from date of surgery to the date of the lowest postoperative BMI. The time until maximum weight regain was calculated from the date of lowest BMI to the date of maximum BMI measured after nadir.

### Nutritional data

Preoperative laboratory values for albumin, calcium, iron, vitamin D, and vitamin B12 were extracted for all patients via the electronic health record. All postoperative values for these labs, drawn within eleven and twenty-five months postoperatively, were also extracted. Nutritional deficiencies were identified in patients for whom resulted laboratory values were less than established criteria for normal range.

### Statistical analysis

All analyses were stratified by surgery type (RYGB or SG). Continuous measures were summarized using means and 95% confidence intervals, or medians and interquartile ranges, and were compared between food security groups using ANOVA or Kruskal–Wallis tests, as appropriate. Categorical patient characteristics were summarized using counts and percentages and were compared using chi-square tests. Associations between food security groups and preoperative BMI were evaluated using the SAS procedure GLM to fit multivariable generalized linear regression models with contrasts statements pairwise comparisons between food security groups. To evaluate differences in postoperative change in BMI, quadratic growth mixed models with time as a quadratic random effect were fit [14, 15]. Contrast statements were written for pairwise comparisons between food security groups. To evaluate the time until BMI nadir and the time until maximum weight regain, Cox Proportional Hazards (PH) Models with contrast statements to test for differences in food security groups were used. The PH assumption was confirmed using Schoenfeld residuals. Finally, to visualize postoperative weight change among food security groups, restricted cubic splines were fit to the data using 5 knots [16, 17]. All models were adjusted for patient preoperative demographic characteristics and comorbidities. All models were robust against adjustment, and tests about food security did not change when non-significant covariates were removed. Results from the full models are reported. All analysis was performed in SAS 9.4 (SAS Institute, Cary, NC). All tests were two-tailed and performed at a significance level of *α* = 0.05.

Geospatial mapping of mRFEI values and percent weight loss following RYGB and SG was completed in R version 4.2.1 (R Core Team, 2022). To perform the mapping, data values were joined to a census tract shapefile of Cuyahoga County from the 2010 decennial Census.

## Results

**Fig. 1:**
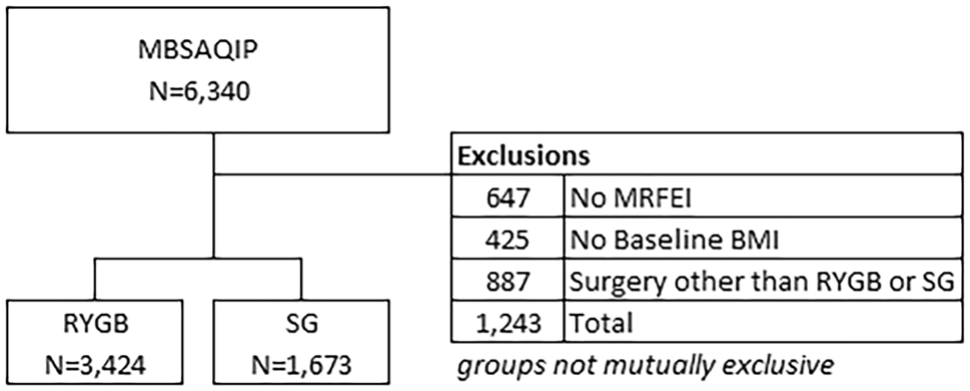
Flow chart for patient inclusion in the study. A total of 5097 patients met the selection criteria. *BMI* body mass index, *MBSAQIP* metabolic and bariatric surgery accreditation and quality improvement program, *mRFEI* modified food retail environmental index, *RYGB* Roux-en-Y gastric bypass, *SG* sleeve gastrectomy

A total of 5097 patients underwent metabolic surgery within our health system from 2010 and 2018 and met the selection criteria in this study (Fig. 1). A total of 3424 patients underwent RYGB and 1673 underwent SG. The median follow-up was 2.3 years (IQR, 0.9–3.6 years). Total weight measurements recorded during four-year follow-up ranged from one to seven, with a median of three follow-up measurements. Weight measurements were distributed uniformly across the follow-up time frame.

### Preoperative characteristics

Seventy-eight percent of patients were female. Median age at the time of surgery was 46.4 years (IQR, 37.6–55.9). Demographic characteristics for each food security group are reported in Table 1 (RYGB) and Table 2 (SG). There were fewer black patients in the F_Secure_ subgroup compared with the F_Swamp_ and F_Desert_ subgroups. There was largely no association between food security, preoperative characteristics, and baseline nutritional variables. The median preoperative BMI was similar across food security groups for both RYGB and SG. There was no association between preoperative BMI and food security for either type of surgery (RYGB *p* = 0.54; SG *p* = 0.70, Tables 1 and 2).

**Table 1:** Descriptive statistics of patients who underwent Roux-en-Y gastric bypass by food security group (based on mRFEI)

Patient characteristic	All patients	Desert	Swamp	Secure	*p*-value
	(*N* = 3424)	(*N* = 900)	(*N* = 885)	(*N* = 1639)	
Age at surgery (years)	46.5 [37.7, 55.6]	46.7 [37.6, 55.9]	46.0 [36.9, 55.7]	46.7 [38.0, 55.3]	0.47^b^
Gender					0.69^c^
Female	2630 (76.8)	699 (77.7)	682 (77.1)	1249 (76.2)	
Male	794 (23.2)	201 (22.3)	203 (22.9)	390 (23.8)	
Preop BMI (kg/m^2^)	45.7 [41.1, 50.9]	45.7 [41.5, 50.8]	45.9 [41.0, 51.0]	45.5 [41.0, 50.8]	0.54^b^
Hispanic					***0.002***^***c***^
Yes	105 (3.1)	21 (2.3)	45 (5.1)	39 (2.4)	
No	3276 (95.7)	866 (96.2)	829 (93.7)	1581 (96.5)	
Unknown	43 (1.3)	13 (1.4)	11 (1.2)	19 (1.2)	
Race*					** < *0.001***^***c***^
Black	734 (21.6)	191 (21.3)	228 (25.9)	315 (19.3)	
White	2581 (75.8)	682 (76.2)	619 (70.3)	1280 (78.5)	
Unk/NR	90 (2.6)	22 (2.5)	33 (3.8)	35 (2.1)	
*Baseline comorbidities*					
Diabetes mellitus					0.10^c^
Yes, non-insulin	795 (23.2)	193 (21.4)	205 (23.2)	397 (24.2)	
Yes, insulin	555 (16.2)	138 (15.3)	131 (14.8)	286 (17.4)	
No	2074 (60.6)	569 (63.2)	549 (62.0)	956 (58.3)	
Severe COPD	68 (2.0)	16 (1.8)	19 (2.1)	33 (2.0)	0.85^c^
Obstructive sleep apnea requiring CPAP/BiPAP	2144 (62.6)	589 (65.4)	550 (62.1)	1005 (61.3)	0.11^c^
Gastroesophageal reflux disease requiring medication	1235 (36.1)	337 (37.4)	309 (34.9)	589 (35.9)	0.53^c^
Hypertension requiring medication	2075 (60.6)	524 (58.2)	533 (60.2)	1018 (62.1)	0.15^c^
Hyperlipidemia requiring medication	1264 (36.9)	303 (33.7)	322 (36.4)	639 (39.0)	***0.03***^***c***^
Renal insufficiency	21 (0.61)	6 (0.67)	4 (0.45)	11 (0.67)	0.77^c^
Currently requiring or on dialysis	19 (0.55)	3 (0.33)	10 (1.1)	6 (0.37)	***0.04***^***d***^
*Baseline laboratory values*					
Preop Albumin* (g/dL)	4.3 [4.1, 4.5]	4.3 [4.1, 4.5]	4.3 [4.1, 4.5]	4.3 [4.1, 4.5]	0.25^b^
Preop calcium* (mg/dL)	9.6 [9.3, 9.9]	9.6 [9.3, 9.9]	9.6 [9.3, 9.9]	9.6 [9.3, 9.9]	0.52^b^
Preop serum creatinine* (mg/dL)	0.81 [0.70, 0.94]	0.80 [0.70, 0.94]	0.81 [0.71, 0.93]	0.81 [0.70, 0.95]	0.53^b^
Preop iron* (µg/dL)	65.0 [51.0, 83.0]	66.0 [51.0, 84.0]	65.0 [51.0, 84.0]	65.0 [51.0, 83.0]	0.86^b^
Preop Vitamin D* (ng/mL)	24.1 [16.4, 32.5]	24.4 [17.2, 32.6]	23.8 [15.7, 32.2]	24.1 [16.5, 32.6]	0.73^b^
Preop Vitamin B12* (pg/mL)	504.0 [378.0, 682.0]	503.0 [377.0, 679.0]	510.0 [379.0, 690.0]	502.0 [376.5, 681.5]	0.82^b^
*Area deprivation Index (ADI)*					
Nationwide ADI Rank	71.0 [49.0, 90.0]	77.0 [50.0, 92.0]	70.0 [50.0, 89.0]	70.0 [47.0, 87.0]	0.045^b^
statewide ADI rank	6.0 [3.0, 9.0]	7.0 [3.0, 9.0]	6.0 [3.0, 8.0]	6.0 [3.0, 8.0]	0.034^b^
*30-Day postoperative outcomes*					
Death	12 (0.35)	4 (0.44)	4 (0.45)	4 (0.24)	0.60^d^
Readmissions	322 (9.4)	88 (9.8)	93 (10.5)	141 (8.6)	0.27^c^
Reoperations	113 (3.3)	32 (3.6)	28 (3.2)	53 (3.2)	0.88^c^

*Data not available for all subjects. Missing values: Albumin = 44; Pre-Op Calcium = 38; Pre-Op Serum Creatinine = 39; Pre-Op Iron = 350; Pre-Op Vitamin D = 534; Pre-Op Vitamin B12 = 339; Race = 19. Statistics presented as median [P25, P75], *N* (column %). *p*-values: b = Kruskal–Wallis test, c = Pearson’s chi-square test, d = Fisher’s exact test

**Table 2:** Descriptive statistics of patients who underwent sleeve gastrectomy by food security group (based on mRFEI)

Patient characteristic	All patients	Desert	Swamp	Secure	*p*-value
	(*N* = 1673)	(*N* = 429)	(*N* = 441)	(*N* = 803)	
Age at surgery (years)	46.4 [37.5, 57.2]	46.2 [37.5, 56.5]	47.2 [37.7, 57.6]	46.5 [37.4, 57.2]	0.76^b^
Gender					0.26^c^
Female	1283 (76.7)	323 (75.3)	330 (74.8)	630 (78.5)	
Male	390 (23.3)	106 (24.7)	111 (25.2)	173 (21.5)	
Pre-Op BMI (kg/m^2^)	45.6 [41.0, 53.2]	45.5 [41.2, 52.3]	45.9 [41.1, 53.1]	45.4 [40.9, 53.6]	0.70^b^
Hispanic					0.63^c^
No	1592 (95.2)	408 (95.1)	425 (96.4)	759 (94.5)	
Yes	53 (3.2)	15 (3.5)	10 (2.3)	28 (3.5)	
Unknown	28 (1.7)	6 (1.4)	6 (1.4)	16 (2.0)	
Race*					0.06^c^
Black	506 (30.3)	135 (31.5)	154 (35.0)	217 (27.1)	
White	1107 (66.3)	280 (65.4)	271 (61.6)	556 (69.3)	
Unk/NR	57 (3.4)	13 (3.0)	15 (3.4)	29 (3.6)	
*Baseline comorbidities*					
Diabetes mellitus					0.62^c^
Yes, non-insulin	304 (18.2)	77 (17.9)	83 (18.8)	144 (17.9)	
Yes, insulin	213 (12.7)	51 (11.9)	65 (14.7)	97 (12.1)	
No	1156 (69.1)	301 (70.2)	293 (66.4)	562 (70.0)	
Severe COPD	64 (3.8)	16 (3.7)	21 (4.8)	27 (3.4)	0.47^c^
Obstructive sleep apnea requiring CPAP/BiPAP	1178 (70.4)	294 (68.5)	326 (73.9)	558 (69.5)	0.16^c^
Gastroesophageal reflux disease requiring medication	472 (28.2)	119 (27.7)	133 (30.2)	220 (27.4)	0.57^c^
Hypertension requiring medication	967 (57.8)	247 (57.6)	265 (60.1)	455 (56.7)	0.50^c^
Hyperlipidemia requiring medication	531 (31.7)	133 (31.0)	148 (33.6)	250 (31.1)	0.63^c^
Renal insufficiency	18 (1.08)	6 (1.4)	5 (1.1)	7 (0.87)	0.65^d^
Currently requiring or on dialysis	26 (1.6)	2 (0.47)	10 (2.3)	14 (1.7)	0.08^c^
*Baseline laboratory values*					
Albumin* (g/dL)	4.2 [4.0, 4.4]	4.2 [4.0, 4.5]	4.2 [4.0, 4.4]	4.2 [4.0, 4.4]	0.21^b^
Preop calcium* (mg/dL)	9.5 [9.2, 9.8]	9.5 [9.2, 9.8]	9.5 [9.2, 9.8]	9.6 [9.2, 9.9]	0.38^b^
Preop serum creatinine* (mg/dL)	0.82 [0.72, 0.97]	0.83 [0.72, 0.96]	0.82 [0.72, 0.97]	0.82 [0.72, 0.98]	0.80^b^
Preop iron* (µg/dL)	64.0 [50.0, 82.0]	65.0 [51.0, 87.0]	62.0 [47.0, 77.0]	65.0 [51.0, 82.0]	0.008^b^
Preop Vitamin D* (ng/mL)	25.0 [16.5, 33.8]	26.4 [17.9, 34.4]	23.7 [15.7, 33.7]	24.7 [16.6, 33.4]	0.12^b^
Preop Vitamin B12* (pg/mL)	508.0 [380.0, 693.0]	508.5 [381.0, 676.5]	519.0 [378.0, 684.5]	505.0 [381.0, 708.0]	0.96^b^
*Area deprivation index (ADI)*					
Nationwide ADI rank	73.0 [54.0, 92.0]	78.0 [56.0, 92.5]	77.0 [55.0, 94.0]	71.0 [50.0, 88.0]	0.002^b^
Statewide ADI rank	6.0 [3.0, 9.0]	7.0 [4.0, 9.0	7.0 [4.0, 9.0]	6.0 [3.0, 8.0]	0.003^b^
*30-Day postoperative outcomes*					
Death	2 (0.12)	0 (0.00)	1 (0.23)	1 (012)	0.99^d^
Readmissions	66 (3.9)	14 (3.3)	25 (5.7)	27 (3.4)	0.10^c^
Reoperations	17 (1.02)	1 (0.23)	6 (1.4)	10 (1.2)	0.15^d^

*Data not available for all subjects. Missing values: Albumin = 89; Pre-Op Calcium = 90; Pre-Op Serum Creatinine = 89; Pre-Op Iron = 209; Pre-Op Vitamin D = 287; Pre-Op Vitamin B12 = 200; Race = 3. Statistics presented as Median [P25, P75], *N* (column %). p-values: b = Kruskal–Wallis test, c = Pearson's chi-square test, d = Fisher's Exact test

### Postoperative change in BMI

**Fig. 2:**
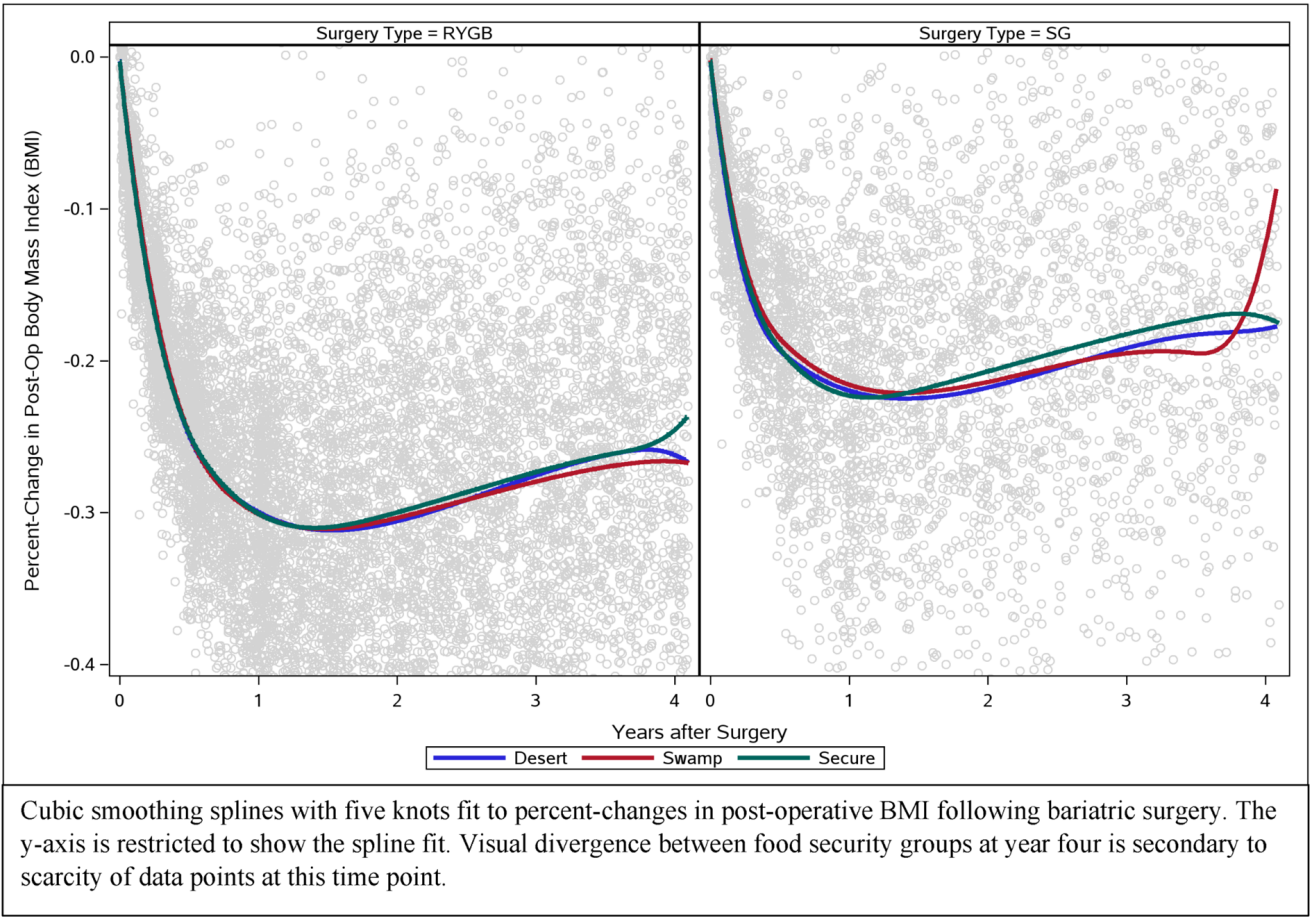
Smoothed trends in weight change after gastric bypass and sleeve gastrectomy stratified by food security status. Cubic smoothing splines with five knots fit to percent-changes in postoperative BMI following metabolic surgery. The *y*-axis is restricted to show the spline fit. Visual divergence between food security groups at year four is secondary to scarcity of data points at this time point

**Fig. 3:**
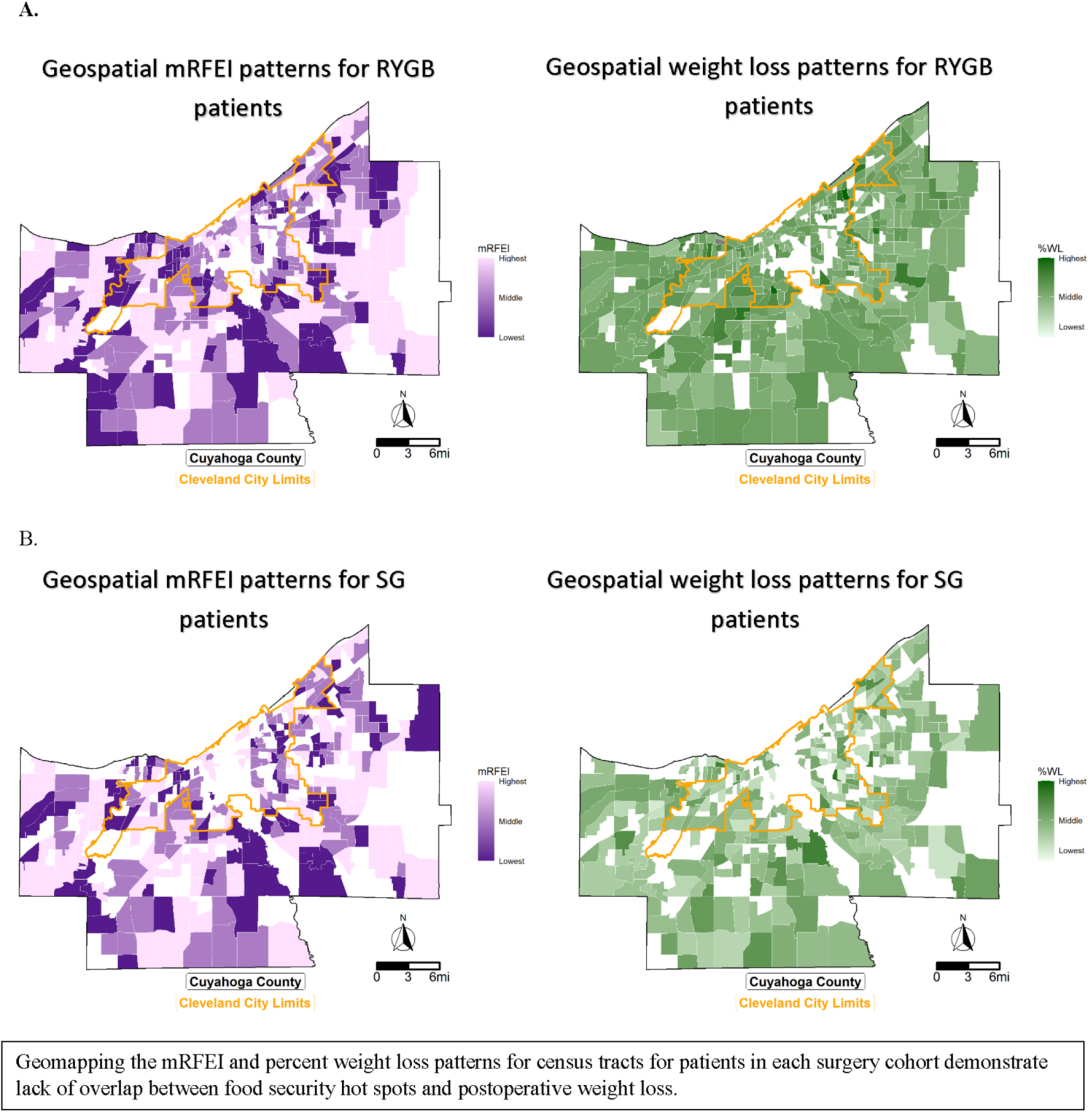
Geospatial mapping of “hot spots” of food deserts and food swamp areas by mREI and geospatial trends in percent weight loss following RYGB (**A**) and SG (**B**). Geomapping mRFEI and percent weight loss for patients in each surgery cohort demonstrate lack of overlap between food security hot spots and postoperative weight loss. *mRFEI* modified food retail environmental index, *RYGB* Roux-en-Y gastric bypass, *SG* sleeve gastrectomy

Food security was not associated with postoperative weight change (RYGB *p* = 0.73, SG *p* = 0.68); Table 3). Cubic smoothing splines fitting percent-changes in postoperative BMI following metabolic surgery were plotted for each food security group and showed similar patterns for percent-change in postoperative BMI across food security groups during a four-year follow-up period (Fig. 2). Finally, for patients residing in the city of Cleveland and, more broadly, Cuyahoga County, there was limited geographic overlap between patterns of food insecurity and percent weight loss following surgery [RYGB (Fig. 3A); SG (Fig. 3B)].

**Table 3:** Differences in preoperative body mass index (BMI) and percent-changes in four-year postoperative BMI after RYGB and SG by food secure groups

Group comparisons	Difference	95% CI: Lower	95% CI: Upper	*p*-value
*Roux-en-Y Gastric Bypass, N = 3279*				
Preoperative BMI				
Secure vs Swamp	0.03	− 0.56	0.63	0.92
Swamp vs Desert	0.15	− 0.53	0.82	0.67
Secure vs Desert	0.12	− 0.47	0.70	0.70
Postoperative percent-changes in BMI				
Secure vs Swamp	− 0.21	− 0.47	0.05	0.12
Swamp vs Desert	− 0.12	− 0.43	0.19	0.46
Secure vs Desert	0.09	− 0.18	0.36	0.51
*Sleeve gastrectomy, N = 1591*				
Preoperative BMI				
Secure vs Swamp	0.01	− 1.11	1.13	0.98
Swamp vs Desert	− 0.12	− 1.40	1.16	0.86
Secure vs Desert	− 0.13	− 1.26	1.00	0.82
Postoperative percent-changes in BMI				
Secure vs Swamp	− 0.11	− 0.50	0.27	0.57
Swamp vs Desert	− 0.20	− 0.64	0.24	0.38
Secure vs Desert	− 0.09	− 0.46	0.29	0.64

Preoperative estimates calculated from generalized linear model estimating preoperative Body Mass Index (BMI). Postoperative estimates calculated from multivariable quadratic mixed effects growth models estimating the percent-change in postoperative BMI. mRFEI = Modified Retail Food Environment Index. Models adjusted for age at surgery, gender, race (Black, White, Other), ethnicity, diabetes, hypertension, dialysis, and CPAP/BiPAP machine use. *P*-values from overall test about food security: RYGB: *p* = 0.90 (pre-op), *p* = 0.73 (post-op). SG: *p* = 0.97 (pre-op), *p* = 0.68 (post-op). Tests were robust against adjustment

### Weight loss nadir

Weight loss nadir occurred at a mean of 1.5 years after surgery for RYGB and 1.3 years postoperatively for SG. Most patients (82.2%) reached their weight loss nadir within 2 years. The overall median BMI nadir of 32 kg/m^2^ was achieved in each group: F_Secure_ (32.1, IQR 27.9–37.7), F_Swamp_ (32.0, IQR 28.0–37.1), F_Desert_ (32.1, IQR 28.0–37.2). There was no association between BMI nadir and food security group (RYGB *p* = 0.60, SG *p* = 0.79). Results by surgery type are reported in Table 4. After adjusting for preoperative factors (sex, race, ethnicity, hypertension, and sleep apnea), the only statistically significant differences in weight loss nadir were seen in F_Secure_ patients who underwent SG; these patients reached their weight loss nadir at slower rates compared to F_swamp_ patients (*p* = 0.02) at the time of surgery. Results were robust against adjustment for additional demographic factors.

**Table 4:** Evaluation of postoperative BMI outcomes (nadir and regain) by surgery type and food security groups

	BMI at nadir				BMI at max regain			
	*N*	Med	IQR		*N*	Med	IQR	
			25%ile	75%ile			25%ile	75%ile
*RYGB*								
All patients	2838	31.1	27.3	35.4	1597	33.8	29.7	39.0
Food desert	754	31.2	27.5	35.3	435	34.3	29.6	38.9
Food swamp	738	30.9	27.0	35.2	423	33.5	29.6	39.0
Food secure	1346	31.0	27.3	35.6	739	33.6	29.7	39.1
*SG*								
All patients	1349	35.3	30.8	41.4	683	38.7	33.9	45.9
Food desert	352	35.3	30.7	41.4	175	38.8	33.9	45.0
Food swamp	355	35.1	30.9	40.7	181	38.8	33.4	44.9
Food secure	642	35.5	30.8	41.5	327	38.5	34.3	46.1

	%WL at weight nadir				%WG at max regain			
	*N*	Med	IQR		*N*	Med	IQR	
			25%ile	75%ile			25%ile	75%ile
*RYGB*								
All patients	2838	30.9	24.6	37.6	1597	9.3	4.9	15.3
Food desert	754	31.4	24.7	37.6	435	9.3	5.2	15.4
Food Swamp	738	31.5	25.2	37.9	423	9.4	4.6	16.2
Food Secure	1346	30.2	24.1	37.5	739	9.1	5.0	15.2
*SG*								
All patients	1349	22.1	16.6	28.4	683	8.9	4.3	13.8
Food desert	352	22.7	16.9	28.5	175	8.4	4.0	15.0
Food swamp	355	22.1	16.6	28.7	181	9.2	5.0	13.9
Food secure	642	21.7	16.6	28.2	327	8.5	4.4	13.5

	HR for time until WL nadir				HR for time until max WG			
	HR	95% CLs		*p*-value	HR	95% CLs		*p*-value
*RYGB*								
Secure vs Swamp	0.96	0.88	1.05	0.41	1.00	0.88	1.12	0.96
Swamp vs Desert	1.06	0.95	1.17	0.31	1.01	0.88	1.15	0.93
Secure vs Desert	1.10	1.00	1.20	0.05	1.01	0.89	1.14	0.88
*SG*								
Secure vs Swamp	0.86	0.75	0.98	*0.02*	1.13	0.93	1.36	0.21
Swamp vs Desert	0.89	0.76	1.03	0.12	0.98	0.80	1.22	0.89
Secure vs Desert	1.03	0.90	1.18	0.63	0.87	0.73	1.05	0.16

*WL* weight loss, *WG* weight regained. Tests about differences in BMI nadir, max regain, %WL at nadir, and %WG at max regain between food secure groups were not significant (RYGB *p* = 0.60, 0.69, 0.08, 0.93; SG *p* = 0.79, 0.89, 0.58, 0.85, respectively). Hazard ratios were calculated from Cox PH models and were reduced using single backwards elimination to test for changes in the association between food security and post-op %WL and %WG. The reduced model adjusted for gender, race, ethnicity, hypertension, and CPAP/BiPAP machine use (selected from univariable analysis with significance *p* < 0.20)

Higher hazard ratios (HR) translate to faster rate of weight loss until nadir is reached

### Weight regain following nadir

The median percent weight regain after weight loss nadir was 9.3% in RYGB patients (IQR 4.9–15.3%) and 8.9% in SG patients (IQR 4.3–13.8%). There was no association between weight regain and food security group (RYGB *p* = 0.93, SG *p* = 0.85). There was also no association between time until maximum weight regain after nadir and food security. Weight regain results were also robust against adjustment for additional demographic factors (Table 3).

### 30-Day postoperative outcomes

There was no association between food security and 30-day readmission (RYGB *p* = 0.27, SG *p* = 0.10), reoperation (RYGB *p* = 0.88, SG *p* = 0.15), or mortality (RYGB *p* = 0.60, SG *p* = 0.99) (Table 1 and 2).

### Nutritional deficiencies

There were no differences in nutritional deficiency rates for albumin, calcium, iron, vitamin B12, and vitamin D by food security group in both patients who underwent RYGB and SG surgery (Table 5). There were no notable differences in missingness of lab data between food security groups.

**Table 5:** Nutritional deficiencies within the first 2 years after metabolic surgery stratified by surgery type and food security groups

Procedure	Deficiency	Lab N	Missing *N* (%)	Food desert	Food swamp	Food secure	*p*-value
RYGB	Albumin < 3.5 g/dL	2143	1281/3424 (37%)	181/592 (31%)	185/575 (32%)	352/976 (36%)	0.06
	Albumin < 2.8 g/dL (Severe)	2143	1281/3424 (37%)	157/592 (27%)	161/575 (28%)	309/976 (32%)	0.07
	Calcium < 8.4 mg/dL	2198	1226/3424 (36%)	84/612 (14%)	80/589 (14%)	147/997 (15%)	0.76
	Iron (< 59 µg/dL)	1626	1798/3424 (53%)	135/458 (29%)	134/433 (31%)	231/735 (31%)	0.77
	Vitamin B12 < 230 pg/mL	1723	1701/3424 (50%)	95/479 (20%)	75/459 (16%)	139/785 (18%)	0.37
	Vitamin D ≤ 20 ng/mL	1730	1694/3424 (49%)	86/474 (18%)	82/461 (18%)	129/795 (16%)	0.63
SG	Albumin < 3.5 g/dL	990	683/1673 (41%)	82/268 (31%)	85/270 (31%)	150/452 (33%)	0.75
	Albumin < 2.8 g/dL (Severe)	990	683/1673 (41%)	63/268 (24%)	68/270 (25%)	129/452 (29%)	0.30
	Calcium < 8.4 mg/dL	1058	615/1673 (37%)	39/286 (14%)	48/288 (17%)	70/484 (14%)	0.56
	Iron (< 59 µg/dL)	682	991/1673 (59%)	63/191 (33%)	52/179 (29%)	107/312 (34%)	0.48
	Vitamin B12 < 230 pg/mL	712	961/1673 (57%)	29/199 (15%)	26/183 (14%)	42/330 (13%)	0.81
	Vitamin D ≤ 20 ng/mL	718	955/1673 (57%)	22/197 (11%)	31/190 (16%)	49/331 (15%)	0.32

Sample sizes: RYGB = 3424, SG = 1673. Missing N and percent are those participants included in this study who had no follow-up between 11–25 months post-op. All other participants had at least one follow-up lab. Sample sizes within food areas varied by lab

### Sensitivity analysis

Following the calculated weight nadir based on available date, 1240 (44%) of RYGB patients and 665 (49%) of SG patients were lost to follow-up (LTF). A sensitivity analysis was conducted to evaluate differences in the patients utilized in the evaluation of weight regain versus those LTF. For both procedures, patients LTF were slightly younger (RYGB: 46 vs 47.9 years, *p* = 0.001; SG: 46.3 vs 48 years, *p* = 0.004). There were no differences between those who were followed and patients who were LTF by food security group (RYGB, *p* = 0.38; SG, *p* = 0.92).

## Discussion

In the most recent statement by the American Society for Metabolic and Bariatric Surgery (ASMBS) and International Federation for the Surgery of Obesity and Metabolic Disorders (IFSO) on the indications for metabolic and bariatric surgery, food insecurity was highlighted as an important stressor that should be identified preoperatively given its potential to significantly impact postoperative outcomes [18]. However, very little was known about the degree to which food insecurity impacts weight loss outcomes after metabolic surgery, particularly SG and RYGB. Given that food insecurity poses a barrier to healthy food choices and is known to impede the impact of medical weight loss programs, we hypothesized that food insecurity would equally and negatively impact outcomes after SG and RYGB.

Our study demonstrated that food security status did not alter postoperative weight change after RYGB or SG. Food security neither impacted weight loss nadir, nor weight regain after nadir for either procedure. Furthermore, food security had no noticeable impact on 30-day readmission, reoperation rate, or mortality. Even more surprising was that regardless of the availability of healthy food options, food security also had no impact on key nutritional outcomes in our study. Deficiencies in albumin, vitamin D, iron, vitamin B12, and calcium were not significantly different between food security groups up to 2 years after surgery, when most weight loss occurred. Thus, these results suggest that both RYGB and SG can be safely and effectively used to treat obesity in patients facing food insecurity.

In reviewing our data, we assessed whether mRFEI scores were associated with area deprivation indices. The area deprivation index, of note, is an extremely well-validated measure established by the Health Resources & Services Administration which assigns national and state-level ranks to neighborhoods by socioeconomic deprivation or disadvantage. It incorporates domains such as income, education, employment, and housing quality [19, 20]. It is currently the most validated scientific tool for neighborhood-level disadvantage among US neighborhoods [21]. Since factors that drive food insecurity include poverty and unemployment [22], we felt ADI may effectively characterize the socioeconomic disadvantage which drives food insecurity on both the geospatial and personal level. In investigating an association between mRFEI and area deprivation index within our data, we found that areas classified as Food Deserts had higher deprivation rates (*p* = 0.045 for RYGB, *p* = 0.002 for SG). Similar to our results looking at metabolic surgical outcomes by mRFEI, we found that national ADI rank was also not associated with changes in post-op BMI, whether or not we adjusted for mRFEI.

Our findings are in line with the few studies that have also sought to evaluate similar questions. Mathson et al. studied the impact of food insecurity on early postoperative outcomes after metabolic surgery. While there was an association between food insecurity and longer length of stay, there was no impact on 30-day mortality [23]. Furthermore, a four-year study of an Appalachian state bariatric program demonstrated that food accessibility did not impact the weight lost at one-year, again highlighting the non-inferiority of metabolic surgery in subjects experiencing food insecurity [8]. To our knowledge, the current study represents the largest study with the longest follow-up time to date on this topic and reports the most comprehensive weight loss outcomes, measuring both weight loss nadir and weight gain after nadir. Another strength of our work is the use of the CDC’s definitions for food insecurity via mRFEI scores, making this work both reproducible and relevant to national policy discussions regarding the care of patients with obesity.

As expected, given the history of Cleveland, Ohio, and the impact of redlining on racial health disparities [24–26], fewer Black patients lived in areas identified as F_Secure_. In one of the largest studies of outcomes of metabolic surgery, African Americans had worse outcomes, including higher mortality rates regardless of whether RYGB or SG had been performed [27]. Though factors underlying these disparate outcomes warrant more study, geospatial disparities in food security do not appear to play a significant role.

## Limitations

This study has several limitations. First, there are well established limitations to the mRFEI as a tool for characterizing food security, such as imprecision of retailer categories used to calculate proportion of healthy food options available within a census tract [28]. Nevertheless, it remains a widely accepted and accessible assessment tool, which increases replicability of our study methods. Second, there was a high number of patients lost to long-term follow-up. However, our sensitivity analysis showed that there were no differences in food insecurity status or relevant cofactors including race or comorbidities between those studied and those LTF. Thus, it is unlikely that those LTF influenced our data or the conclusions of this study. Third, a minority of patients moved during the study period, and thus may have switched food security groups. [29]. We reviewed our data and found that 14.5% of RYGB patients (85.5% were in the same zip code) and 12% of SG patients (88% stayed within the same zip code) moved to a different zip code within 2 years of their index metabolic surgical procedure. Fourth, food insecurity represents a significant stressor which can impact psychological outcomes after metabolic surgery, including depression, weight-based discrimination, and poor self-image, all of which can impact quality of life postoperatively [30]. This was not addressed in our study and remains an area of future study. Fifth, this study only addresses the impact of geospatial food insecurity on metabolic surgery outcomes from a population standpoint and does not address factors related to personal food insecurity, such as access to transportation, food assistance programs such as the Supplemental Nutrition Assistance Program (SNAP), or community food programs. This represents an area of interest for future study. Sixth, this study was a retrospective analysis at a single center, with geographic study area limited to the northeast Ohio region and demographics. Thus, further work in a variety of community settings is warranted to confirm our findings. Finally, it is a possibility that the rigorous assessment and patient selection process for metabolic surgery in and of itself inadvertently excludes some of the most socially and economically vulnerable patients of society and may select for relatively less socioeconomically disadvantaged patients, even among those living within food insecure areas. This is a potential reality of which surgeons should be cognizant and should prompt strategies for improved access to metabolic surgery for all patients.

## Conclusion

In conclusion, our work suggests that food security status may not significantly impact weight loss or nutritional outcomes following metabolic surgery. In fact, our data shows that even for those living in food insecure areas, desired weight loss can be achieved postoperatively and maintained at expected levels. Despite the association of food insecurity and obesity, food insecurity status should not preclude patients from eligibility for metabolic surgery. Metabolic surgery may offer one of the most most equitable solutions currently available to address the complex and ever challenging obesity epidemic in the United States.
